# Fabry disease: patients at risk in Brazil!

**DOI:** 10.1590/2175-8239-JBN-2021-E002

**Published:** 2021-02-10

**Authors:** Hugo Abensur

**Affiliations:** 1 Universidade de São Paulo, Faculdade de Medicina, Hospital das Clínicas, Beneficência de São Paulo, São Paulo, SP, Brasil.

Fabry disease (FD) is caused by deficiency or absence of α-galactosidase A activity, which is a lysosomal enzyme, which substrate is globotriaosylceramide (GB3 or GL-3). Therefore, in this disease, GB3 builds up in the lysosomes of all cells of the human body, compromising the functions of several organs. FD has inheritance link to the X chromosome, it affects all ethnicities and has an incidence not well determined, between 1/100,000 to 1/500,000 inhabitants. In addition, more than 600 mutations have been described in the α-galactosidase A^1^ gene. The clinical picture is highly variable, depending on the type of mutation and the gender of the patients. In the classic form, which affects male children, the clinical picture is exuberant, with the presence of angiokeratomas, corneal deposition, acroparesthesia, heat intolerance; over the years, kidney, cardiac and gastrointestinal disorders occur. In patients with less severe mutations and in women, the onset of manifestations is later, and it often affects only one organ^2^.

The diagnosis is based on the clinical picture, family history and active search for the disease in risk groups (screenings), such as patients on dialysis, patients with myocardial hypertrophy without apparent causes and patients with early strokes^3^. In this edition, Sodré L et al.^4^ evaluated relatives of 71 patients with FD mutation, detected among 36,442 dialysis patients in a previous study. From these 71 patients, 1,214 possible CF patients were detected by medical anamnesis. In these patients, the activity of the α--galactosidase A enzyme was evaluated, and in cases that showed changes, a genetic study was carried out to confirm the FD mutation. Finally, 115 patients with the FD mutation (9.47%) were detected, 66.1% women and 74% under 44 years of age. Therefore, through the screening performed it was possible to find a very large number of patients with this rare disease. The advantages of this approach are that it enables the early diagnosis and the establishment of specific treatments before the affected organs are severely compromised.

The treatment of FD consists of enzyme replacement therapy (ERT) with recombinant α--galactosidase A enzymes in order to avoid or remove GB3 intracellular deposits. In addition, the patients should receive specific treatments for the involvement of different organs, such as antiproteinuric drugs, angiotensin converting enzyme inhibitors or angiotensin II receptor blockers, in the case of renal impairment. More recently, a new approach to certain types of less serious mutations has become available in Brazil. A drug, offered orally, which stabilizes the altered enzyme (chaperone), returns the functionality of the enzyme. According to an European guideline, in men with the classical form, ERT is recommended as soon as there are clinical signs of renal, cardiac or cerebral involvement, but it can be considered in patients aged ≥ 16 years, in the absence of clinical signs or symptoms of organ involvement. Women and men with non-classical FD should be treated as soon as there are early clinical signs of kidney, cardiac, cerebral and gastrointestinal involvement.^5^

However, the availability of these treatments for patients with FD in Brazil is being threatened. The preliminary report from the National Commission for the Incorporation of Technologies to SUS (CONITEC) found that the available scientific evidence on the use of enzyme replacement therapy with alpha-agalsidase or beta-agalsidase in patients with Fabry disease does not demonstrate benefit in important clinical outcomes, or modification of the natural course of the disease. In addition, the best available evidence is limited as to the number of patients included, and a short follow-up time considering the chronicity of the disease and the continuous use of medications. There is also the major budget impact that adding it would represent to SUS (Brazilian Public Healthcare System). This report was submitted to public consultation, which ended on 09/14/2020.

FD is devastating and deserves treatment directed at its original defect, which is the lack or deficiency of the α--galactosidase A enzyme. Type 1 diabetes is not treated with symptomatic drugs, but with the replacement of the missing insulin, which is offered by the SUS to patients. The kidney disease anemia, caused by the deficiency of a hormone, erythropoietin, is also treated with the replacement of recombinant human erythropoietin, which is also offered by the SUS.

Unlike diabetes, FD is a rare disease, which makes it difficult to demonstrate the therapeutic efficacy of existing treatments. In the CONITEC assessment, only randomized studies were included. Mechanistic studies, an important tool for evaluating therapeutic efficacy in rare diseases, were not considered. In randomized studies, patients already at an advanced stage of the disease were included, which makes it difficult to demonstrate the effectiveness of enzyme replacement in organs that are already irreversibly compromised.

The hope is that, with this public consultation, this position from CONITEC will be changed, for the good of patients with FD in Brazil.
